# Constipation in Ulcerative Colitis: An Underestimated Problem

**DOI:** 10.3390/jcm14155428

**Published:** 2025-08-01

**Authors:** Gabrio Bassotti, Sara Bologna, Elisabetta Antonelli

**Affiliations:** 1 Gastroenterology, Hepatology and Digestive Endoscopy Section, Department of Medicine and Surgery, University of Perugia, 06123 Perugia, Italy; 2 Gastroenterology and Hepatology Unit, Perugia General Hospital, 06121 Perugia, Italy; sara.bologna@ospedale.perugia.it (S.B.); antelibetta@yahoo.com (E.A.)

**Keywords:** colon, colon motility, constipation, inflammatory bowel diseases, ulcerative colitis

## Abstract

Ulcerative colitis is a chronic intestinal disorder that belongs to the category of inflammatory bowel diseases, and is usually characterized by the presence of bloody diarrhea and abdominal pain, due to an accelerated transit and intestinal sensibilization following inflammation of the colonic mucosa. However, the literature reports that ulcerative colitis may sometimes feature fecal stasis with constipation. This apparent paradox may be partially explained by the motor abnormalities of the large bowel following inflammation, damage to the enteric innervation, and the onset of parietal fibrosis over time. Moreover, some anorectal abnormalities such pelvic floor dyssynergia may explain the symptoms of constipation reported in subsets of patients. Since these abnormalities may be responsible for diagnostic delays and non- or partial responses to therapy, it is important to recognize them as early as possible to avoid incorrect clinical and therapeutic approaches to these patients.

## 1. Introduction

Ulcerative colitis (UC) belongs to the category of inflammatory bowel diseases (IBD), and is characterized by diffuse inflammatory aspects of the large bowel mucosa, extending from the rectum. The main clinical manifestations are abdominal pain, diarrhea with rectal bleeding, weight loss, and other systemic symptoms such as fever [1]. Although mucosal inflammation is traditionally considered to involve only the colonic mucosa and submucosa, there is increasing evidence that, especially in a subgroup of patients with long-standing disease, the inflammatory state may deepen and cause progressive fibrosis of the large bowel [2]. These abnormalities, in turn, reflect on colonic function, and especially on its motor activity, as discussed below. Thus, it is not surprising that the clinical effects of inflammation, such as erosions, ulcers, and mucosal bleeding, may cause diarrhea as a main symptom, often associated with abdominal pain. However, UC should be considered a multi-faceted clinical entity, and there is evidence in previous studies that patients with this condition may paradoxically present to physicians as fecal stasis and complaints of constipation [3,4]. Due to its somewhat low frequency, this aspect has been relatively neglected by researchers for years, but it has resurfaced more recently in the literature [5], and it has also been regarded as a special topic in consensus conferences on UC [6,7]. In this article, we will consider and discuss the presence of fecal stasis and its clinical consequences, such as constipation, in patients affected by UC.

## 2. Fecal Stasis and Constipation in UC

It is interesting to note that some of the first reports in the literature concerning the presence of constipation in patients with UC were published in the 1960s/1970s [3,4]. After an early description of six cases of proctocolitis investigated by radiological means (barium enema) showing disease activity in the left colon with barium retention in the right colon [3], the presence of fecal stasis was subsequently reported in about 10% of a series of patients with UC [4]. In that study, in which the patients were investigated using a combination of radiological methods associated with radiotelemetry, the authors showed that the patients with fecal stasis had hypotonia of the proximal colon associated with luminal dilatation [4]. However, these studies were carried out using suboptimal methods (barium enemas and radio telemetric capsules, which do not adequately detect motor abnormalities of the large bowel), which somewhat limited the actual value of the observations.

A subsequent study, limited to a clinical assessment, reported that approximately one-third (27%) of patients with UC included in the assessment evacuated hard stools, indicative of constipation. These symptoms were found more frequently in patients with active UC, and the authors concluded that this subset of patients suffered from proximal constipation and distal colonic irritability [8]. A further study published in 1991 investigated the whole colonic transit in patients with UC using an objective technique (radiopaque markers), and demonstrated that transit was delayed in 11% of patients. The markers were distributed throughout the colon, and the authors concluded that approximately 10% of attacks of distal colitis might be associated with fecal stasis [9]. These attacks were correlated by the investigators to “proximal” fecal stasis accumulating in the uninflamed colon above an area of active colitis.

In the following years, the issue of constipation in patients with UC almost disappeared in the literature until 2013, when some authors reported the presence of abnormal defecation dynamics (pelvic floor dyssynergia), documented by manometric techniques, in a small cohort of patients with IBD complaining of persistent defecatory problems and constipation despite clinical improvement. This cohort included a small group of patients with UC (with six subjects). Based on these observations, the authors suggested that the presence of disordered defecation and constipation after the inflammatory state had abated should prompt specific investigations to diagnose these potentially treatable abnormalities [10]. However, the number of patients with UC was too small to draw firm conclusions from the study. Similar results were subsequently reported in another study in which the authors investigated 38 patients with UC and showed that a relevant proportion (approximately 40%) of these patients complained of constipation, with symptoms likely related to abnormal defecation dynamics [11].

A more precise definition of “proximal constipation”, based on more objective criteria derived from the Rome criteria for functional constipation (widely validated and well-accepted in the literature), was eventually provided in the study of James at al [12]. In this study, proximal constipation in patients with UC was considered as the presence of at least two of the following symptoms: bloating, excessive or troublesome wind, abdominal cramping pain, reduced frequency of defecation compared with patients’ own frequency, passage of hard or dry stools, straining at stool, and sensation of incomplete defecation. These symptoms needed to be present for at least three days per month during the previous three months [12]. The study, carried out in more than 100 patients with UC, revealed that approximately 50% of the patients fulfilled the definition of proximal constipation, which was associated with female sex and left-sided active disease, but not with age, disease duration, or therapy. Compared to those without the condition, most patients with proximal constipation underwent an increase in the dosage of anti-inflammatory therapies and the use of laxatives and fiber supplements [12].

Other authors evaluated the effectiveness of behavioral treatment in patients with IBD in a later study, which included a small subgroup of 12 patients with UC presenting with ongoing symptoms of constipation or fecal incontinence despite adequate therapy, and showed that this approach may be beneficial in more than 80% of constipated subjects [13].

More recently, the aspects of constipation associated with UC have been investigated by Japanese authors using more precise criteria to define constipation, i.e., those described in the Rome working teams’ reports. In the first study on approximately 300 patients with UC, the authors reported that the prevalence of constipation (evaluated according to Rome I criteria) was 12.4%, and that the symptoms were associated with the severity of nocturia [14]. In a second study, the same group of investigators reported that aging was independently and positively associated with the prevalence of constipation. The latter was not associated with clinical remission, mucosal healing, duration of UC, or extent of the disease [15]. In a third study on almost 400 patients with UC, the prevalence of constipation was 12.5%, and it was not associated with the presence of allergic diseases [16]. However, these were all clinical investigations based on self-administered questionnaires, and no mention of the use of large bowel transit was made; therefore, we do not know whether “proximal constipation” played a role in these patients.

Finally, we must not forget that the incidence of UC is not rare in pediatric patients [17], and that this pathological condition is rising globally [18]. Thus, it is likely that in pediatric patients, the issue of constipation associated with UC might also be of some clinical relevance. Unfortunately, such studies are infrequent in the literature, and only one recent investigation has been published on this topic. In this study, conducted on a cohort of 238 children affected by IBD (130 of whom, 56%, were affected by UC), approximately 20% of them complained of constipation [19]. Overall, compared with non-constipated children, those with constipation presented with a significant diagnostic delay of IBD. Notably, constipated patients with UC displayed a more limited disease involvement than patients affected by Crohn’s disease, with a significantly frequent anatomical localization observed in the rectum (30%) and in the left colon (15%).

## 3. Constipation in UC: A Challenging Definition

Based on the above considerations, there are some doubts that a major issue in defining constipation in patients with UC may be related to the fact that the term “proximal constipation” is not universally accepted in the literature, and its connotations may be misleading. Although the term “constipation” is generally understood, the adjective “proximal”, even if supported by some evidence in the literature, poorly defines the problem, and might not be representative of all constipation symptoms occurring in patients with UC. As constipation can occur in patients with pancolitis and those with distal disease, the term ‘proximal constipation’ may be considered an inaccurate representation of the underlying situation.

In addition, as reported above, a certain number of patients with UC display constipation symptoms related to abnormal dynamics of the anorectal area, and therefore cannot be considered as “proximally constipated”. Thus, replacing this obsolete and inaccurate term with a more precise nomenclature for this clinical syndrome could lead to a more precise identification of the problem and to the development of enhanced treatment modalities. Therefore, the term “ulcerative-colitis-associated constipation syndrome” (UCAC) might provide a more accurate description of this clinical scenario. This term, adapted for patients with UC and featuring symptoms of constipation, is based on the simplification of some definitions already adopted and validated for the Rome criteria. In fact, the use of the term UCAC would allow patients with any severity of disease to be included, since it makes no mention of disease activity, and indicates the relationship between the two disorders, rather than defining causality [12]. Unfortunately, this term has not yet been systematically adopted when dealing with constipation in UC, as can be seen in recent studies [13,14,15,16,19]. Another limiting factor of this approach is that objective investigations of transit assessment are not required to support the diagnosis. The absence of both a widely accepted definition and a suitable methodology for the study of this population, as well as the difficulty of studying this patient subcohort, may be the reasons why the issue of “proximal constipation” in patients with UC has received very little attention in the published literature. Consequently, the true prevalence of this condition and the most effective therapeutic interventions are still poorly defined.

## 4. Pathophysiological Considerations

### 4.1. Colonic Motility

In humans, the motility of the large bowel is characterized by several complex contractile activities, which are strictly linked to each other and are—mostly—controlled by the enteric nervous system. These activities are mainly represented by low- and high-amplitude propagated contractions (LAPC and HAPC, respectively) [20,21], cyclic motor patterns [22], and simultaneous pressure waves [23], occurring as single phenomena or arranged in patterns. These patterns widely fluctuate during the day, according to a circadian trend that displays its maximum grade of activity during the daylight hours [24], and are influenced by some common daily physiological events.

The most powerful physiological events that elicit colonic motor activity are represented by meals [25], awakening (especially in the morning), and endoluminal distension. The colonic contents then reach the rectum more or less rapidly under the influence of the above motor patterns, thus eliciting the distension of the rectal ampulla. Therefore, the parietal receptors of the rectum and the sampling reflex of the anal sphincter send the relevant stimuli to the brain, and if socially appropriate, the subject will decide whether to defecate [26].

### 4.2. Effects of Inflammation on the Colon

As shown by experimental and clinical evidences, the mucosal inflammation documented in UC patients has several deleterious influence on colonic homeostasis, including impairment of mucus production [27] (with subsequent possible increase in motility-induced shear stress [28]) and derangement of colonic innervation [29]. The latter is particularly important from a pathophysiological perspective, as it controls the movements of the viscus through its intricate neural networks. Several abnormalities of the enteric nervous system have been repeatedly documented in patients with UC, which involve almost all cell types present in the enteric ganglia [30,31]. These findings are also supported by experimental animal models of colitis, showing that inflammation directly or indirectly affects the nervous and muscular components of the large bowel wall, and may also alter enteric neurotransmission [32,33,34,35,36,37], in turn influencing anorectal and colonic motility [38,39]. As a result, the latter is frequently abnormal in patients with UC [40]. Unfortunately, most of these studies have been carried out in patients with active disease, clinically presenting with bloody diarrhea, and the motility aspects in patients with fecal stasis/constipation have been largely neglected, apart from the anorectal area, which is easier to investigate by manometric techniques (see above).

## 5. Why Are Some Patients with UC Constipated?

**Figure 1:**
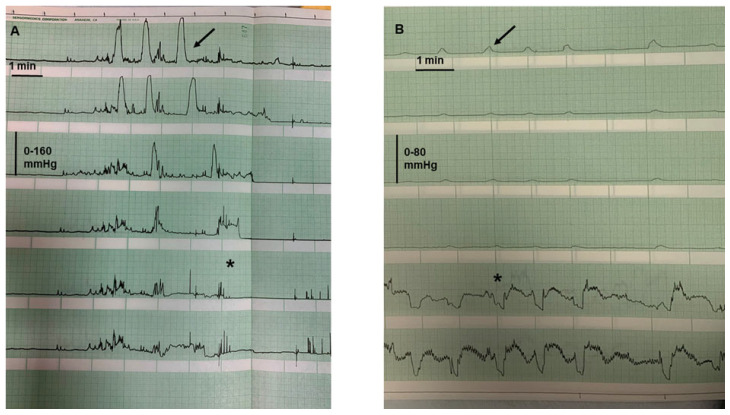
(**A**) Archival manometric tracing of a patient with moderately active UC, showing the presence of high-amplitude propagated contractions (arrow) with concomitant stimulus to defecation (asterisk). Recording points (distanced 12 cm apart) spanning from the mid transverse (first tracing) to the anal sphincter (last tracing). (**B**) Archival manometric tracing of a patient with UC in remission, showing the presence of pancolonic pressurizations (arrow) concomitant with relaxations of the anal sphincter (asterisk). Recording points as in tracing A.

Although, as seen above, the most frequent effect of colonic mucosal inflammation have a clinical phenotypical expression, represented by the presence of bloody diarrhea, and unfortunately, there are no studies available on colonic motility in constipated patients with UC. Some pathophysiological considerations may be made concerning this latter issue. For instance, some studies have demonstrated that in subjects with moderately active UC there is an increased number of HAPC (the manometric equivalent of mass movements, suggesting an increase in propulsive abilities of the colon, likely related to the endoluminal inflammation) [40] (Figure 1A). This phenomenon was not documented in patients with quiescent disease (Figure 1B), suggesting that colonic motility might be decreased and the transit slowed when the inflammation has abated. Moreover, the colonic motor response after eating (represented by an increase in overall colonic motility starting soon after the first mouthfuls and lasting a few hours) may be reduced in intensity and shortened in UC patients compared to controls [41,42], suggesting another possible mechanism of delayed transit.

Some objective demonstration of delayed colonic motor activity (especially in the proximal segments) was also obtained by means of transit studies [9,43,44]. The limited available objective evidence suggests that the mucosal inflammation of the large bowel on one hand may accelerate the transit in the actively affected segments, and on the other hand might slow the progress of colonic contents in the segments proximal to those involved in the inflammatory process [4,9,43,44]. This delay in transit likely happens via inhibitory signals involving the control of large bowel motility, both in the basal state and after consuming a meal. Another contributing factor, often present in patients with long-standing disease, may be due to the onset of fibrosis, initially appearing at mucosal level, which progressively deepens within the colonic wall, and then involves the nerves and muscles of the large bowel. This phenomenon could likely be responsible for the development of an abnormal colonic motility and of delayed transit [2]. Of course, the presence of pelvic floor dyssynergia (documented in a subset of patients with UC [10,11]) may also contribute to slow the transit in proximal segments [45].

## 6. Clinical Consequences

Although only observed in a subset of patients with UC, the presence of constipation may have important clinical consequences [5]. These include the diagnostic delay of the inflammatory condition (as observed in pediatric patients) [19], the persistent disturbances of the defecatory function despite adequate therapy [10,11], and the increased dosages of anti-inflammatory therapies [12].

The latter point is of particular interest, and it is supported by evidence obtained in some investigational reports. The study of Cowan and colleagues has demonstrated that inadequate doses of sulphasalazine in patients with UC may be related to fecal stasis in the proximal colon, which is normalized after treatment with hydrophilic colloids or bran, which accelerates the transit [46]. Further, in another study the regional distribution of Eudragit-coated gelatin capsule showed an exaggeration of the usual asymmetrically distributed delayed release oral formulation in the proximal colon of patients with UC (91%) compared to controls (69%). The authors concluded that these observations suggest a reduced exposure of the distal colon to orally dosed topical agents [47].

The presence of constipation implies that therapeutic actions can often be taken to alleviate symptoms, such as the intensification of anti-inflammatory medications, which may not be exactly appropriate for patients with inactive disease or functional disorders. Laxative use can be an option; however, there are no data on which type of laxative or fiber supplement can be used to avoid toxicity, and on their possible interactions with the drugs used to treat UC.

Furthermore, it is unknown whether prokinetic agents or dietary modifications have any role in this particular clinical subset of constipated patients. These observations, in our view, bear a certain clinical importance, since in patients with UC and constipation, the response to pharmacologic agents may be impaired, with a consequent lack or partial response to the therapy. On the other hand, an early recognition of constipation related to pelvic floor abnormalities may result in a prompt and effective therapeutic approach, such as that represented by biofeedback, likely to be of benefit in a consistent proportion of subjects [48].

## 7. Future Perspectives

There are few doubts that detecting as early as possible the presence of fecal stasis and constipation in patients with UC may represent an important clinical bonus and improve the clinical course and the therapeutic approach. In fact, now, it is very well known that constipation deeply affects the quality of life of patients in general, and represents an important factor of health care utilization. Unfortunately, to date, we have usually had to rely mainly on the clinical aspects (clinical history, physical examination) and on limited instrumental assessments (conventional radiology, anorectal manometry) to diagnose “proximal constipation” in patients with UC.

We feel that it is vital that the first step concerning this issue should be the adoption of a common nomenclature, since the term adopted up to now (i.e., “proximal constipation”) appears confusing and misleading. Under this light, it is our opinion that the use of a more appropriate term, such as UCAC, might be timely and useful, since it provides a more precise definition of the problem, and it is based on some simplified but internationally accepted Rome criteria [12]. Moreover, the adoption of such a definition could at least lead those researchers interested in this aspect to start speaking a common language, as advocated in other fields [49], and improve the communication between physicians and patients [50].

**Figure 2:**
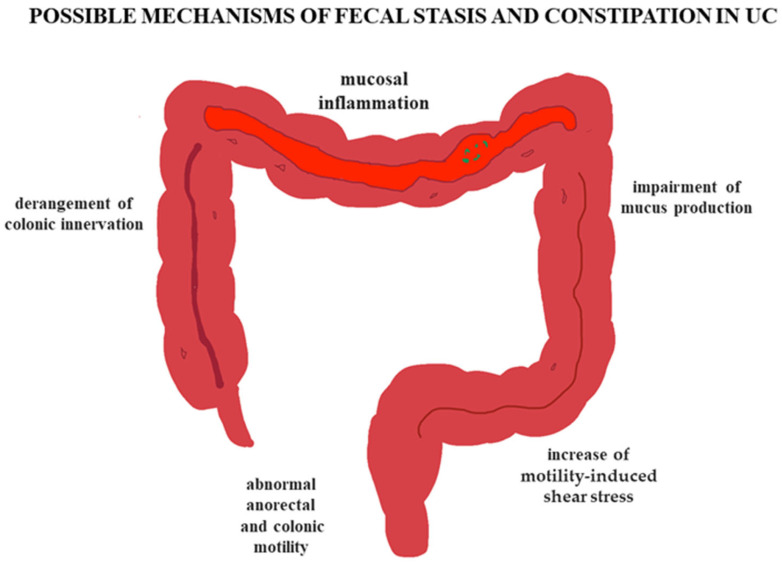
Schematic representation of the possible pathophysiological mechanisms underlying fecal stasis and constipation in patients with ulcerative colitis (UC).

Another important limiting factor to be faced is the still scarce knowledge of the actual pathophysiological mechanisms underlying constipation in patients with UC (Figure 2). In fact, at present, the possible evaluations aimed at studying colonic motility in order to explore its complex patterns, such as pancolonic manometry [51] and colonic scintigraphy [52], are only available in a handful of centers.

Moreover, even when available, these techniques are mainly devoted to research purposes, are more or less invasive, and are limited by an incomplete knowledge of the basic physiologic phenomena. However, on the positive side, there is the fact that technological advances and improvements are progressing and non-invasive methods, in particular those concerning the use of magnetic resonance, are being applied to study gastrointestinal motility [53,54] and colonic motor function [55], even in the pediatric population [56]. Unfortunately, since magnetic resonance imaging for the study of gastrointestinal motility is still in its infancy, it is scarcely available and somewhat expensive for such clinical purposes, even though its lack of invasiveness likely makes it an ideal method to address these issues. The potentialities of this technique, however, are extremely wide, and can offer important insights into several aspects related to colonic motor activity. For instance, in the near-future, experimental models using this technique will hopefully be able to characterize the luminal flow inside the large bowel and to predict the dissolution of pharmaceutical formulations, optimizing the targeted release of substances within the viscus [57].

Finally, we should not forget that patients with UC usually suffer from health-impaired quality of life [58] and that the presence of constipation in these subjects is likely to further decrease it. Indeed, some evidence in the pediatric population suggest that the presence of constipation inpatients with IBD is one of the gastrointestinal symptoms that may predict a worsening of the quality of life in these subjects [59].

## 8. Conclusions

Notwithstanding the scarce knowledge on the presence and on the pathophysiological grounds of fecal stasis and constipation symptoms in patients with UC, this topic has remained relevant over time, and recently there has been a renewed interest in it. Based on the limited evidence available in the literature, we can state that although seemingly paradoxical, it is a real clinical issue; therefore, it must be known, and hence suspected, when dealing with patients affected by UC, especially when in response to a scarce or absent therapeutic response. It is noteworthy that symptoms of fecal stasis and constipation (related to colonic, anorectal, or both abnormalities) can be present during both the florid state and the phases of remission of the disease, and these symptoms can significantly impair the quality of life of patients with UC. In addition, it should always be kept in mind, especially in the pediatric population, that constipation may actually precede the onset of ulcerative proctitis [60].

Until now, the best tool for diagnosing this condition, the optimal treatment including effectiveness in controlling symptoms and quality of life, and the true prevalence of fecal stasis and constipation in UC have remained uncharted, and should be better explored and established in well-planned investigations. There is no doubt that this issue needs more in-depth pathophysiological information and a better clinical definition, and further studies are needed to understand this fascinating clinical conundrum.

## Data Availability

No new data were created or analyzed in this study.

## Abbreviations

The following abbreviations are used in this manuscript:

HAPC	High-amplitude propagated contractions
UCAC	Ulcerative-colitis-associated constipation
IBD	Inflammatory bowel disease
LAPC	Low-amplitude propagated contraction
UC	Ulcerative colitis
